# Rapid electrochemical detection of *Escherichia coli* using nickel oxidation reaction on a rotating disk electrode

**DOI:** 10.1016/j.cej.2021.128453

**Published:** 2021-05-01

**Authors:** Ashwin Ramanujam, Bertrand Neyhouse, Rebecca A. Keogh, Madhivanan Muthuvel, Ronan K. Carroll, Gerardine G. Botte

**Affiliations:** a Chemical and Electrochemical Technology and Innovation Laboratory, Department of Chemical Engineering, Texas Tech University, Lubbock, TX 79409, USA; b Center for Electrochemical Engineering Research, Department of Chemical and Biomolecular Engineering, Ohio University, Athens, OH 45701, USA; c Department of Biological Sciences, Ohio University, Athens, OH 45701, USA

**Keywords:** Electrochemical microbial sensor, Amperometric biosensor, Nickelelectrochemical oxidation, *Escherichia coli* detection

## Abstract

A standalone electrochemical method for detecting the bacterium *Escherichia coli* in water was developed using a nickel electrode and no biorecognition element. Electric current responses from different *E. coli* concentrations were recorded based on their interaction with a locally formed electrocatalyst. A rotating disk electrode was used to minimize the mass transport limitations at the interface. Results from experiments with the rotating disk electrode also paved the way for hypothesizing the detection mechanism. The operating conditions were established for sensing the electric current responses in the presence of *E. coli*. The least-squares linear regression model was fit to the data obtained from currents of some known *E. coli* concentrations. This probe had a detection limit in the order of 10^4^ CFU/ml. The response time to detect the presence/absence of *E. coli* was less than half a second, while the total assay time, including quantification of its concentration, was 10 min. The electric current response from a solution mixed with *E. coli* and *S. aureus* showed current similar to *E. coli* only solution indicating the specificity of the sensor to respond to signals from *E. coli*. This electrochemical microbial sensor's uniqueness lies in its ability to rapidly detect *E. coli* by forming the catalyst locally on demand without the attachment of biorecognition elements.

## Highlights

• A direct (no enzymes needed) electrochemical microbial sensor (EMS) was developed.
• Nickel is used as the electrocatalyst which is converted into active form locally.
• A linear calibration equation was established for *E. coli* detection using the EMS.
• EMS can sense the presence/absence of *E. coli* within half a second.
• Exhibits response specific to *E. coli* in the presence of *S. aureus.*

## Graphical abstract

**Figure:**
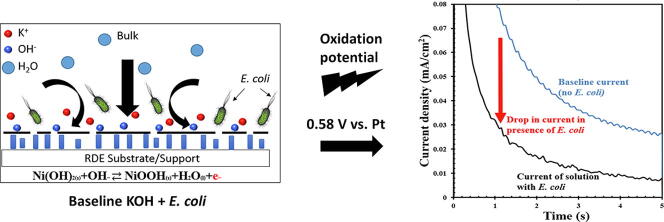

## 1 Introduction

Access to clean drinking water continues to be a significant concern in areas around the globe. According to the drinking water fact sheet released by the World Health Organization (WHO) in June 2019, about 2 billion people consumed water contaminated with feces [1]. Consumption of contaminated water could lead to deadly diseases such as cholera, typhoid, and dysentery. Hence, thwarting this environmental issue by detection of pathogenic bacteria remains a critical component of wastewater treatment. Standard detection methods, such as culturing on selective and differential media or amplification methods such as polymerase chain reaction (PCR) [2], require several hours or days to return satisfactory results. This delay in obtaining results incites a demand for more rapid and more efficient detection methods. Reducing the analysis time, simplified analytical procedure, and selectivity to pathogenic bacteria are vital to improving water quality around the world. Failure to detect and inactivate extremely pathogenic and harmful bacteria can mean life or death for an entire population.

*E. coli* is the standard indicator species used to detect coliforms in water. It has a reasonably short doubling time of 20 min and is usually present if other coliforms are in the water. Even with its short doubling time, growing *E. coli* on media requires overnight incubation for colony counting. Researchers have developed a vast array of sensing methods to reduce the time it takes to count microorganisms, specifically indicator bacteria such as *E. coli*. These methods include but not limited to, colorimetry, bioluminescence, and fluorescence for bacterial enumeration [3], [4]. More recently, however, the use of electrochemical biosensors have been expanded to meet the demand for an accurate and efficient means of detection. The interactions and corresponding responses of *E. coli* with electrodes have widely opened the possibility of using bio electroanalytical devices for their rapid detection [5], [6]. Electrochemical biosensors provide sensitive detection, rapid data acquisition, and relatively simple analytical procedures at an economical cost, making them the ideal candidates for effective detection of microorganisms [3], [7].

A multitude of comprehensive review articles have been published within the scope of electrochemical biosensors over the last several years, detailing their application in the detection of bacteria and viruses as the field has expanded [2], [4], [8], [9], [10], [11], [12]. Electrochemical biosensors employ a wide variety of synthetic techniques and electroanalytical measurements to obtain selective, sensitive, and rapid detection. For example, one common approach that has been reported by several groups is the measurement of intracellular enzymes present in *E. coli* – enzymes such as β-d-glucuronidase (GUS) and β-d-galactosidase (Gal) extracted through enzyme induction, and reactions with various substrates on enzymes form electroactive products that can be measured and correlated to varying concentrations of bacteria using amperometric and potentiometric techniques [13], [14], [15], [16], [17]. Alternatively, other groups have reported using impedimetric sensors where a substrate is chemically adsorbed onto an electrode surface (typically gold) to allow microorganisms to bind to the electrode, creating a complex electrode-solution interface. Subsequently, electrochemical impedance spectroscopy (EIS) can be applied to measure charge transfer resistance, which correlates to varying bacteria concentrations [18], [19], [20], [21]. While many of these methods offer high sensitivity and low detection limits, they often require complex synthetic procedures and sophisticated analytical techniques. These requirements limit the practicality of these technologies on a more global scale. Furthermore, most of these techniques rely on enzymes attached to the electrode surface to interact with target species. If the enzymes are inactivated due to variation in pH or temperature, which is likely to occur in a field scenario, the sensor would inevitably lose its functionality. Typically, the lifetime of an enzymatic biosensor is limited to 2–8 weeks [22]. Thus, a long-lasting electrochemical biosensor utilizing easily accessible materials and limited electrode modifications would advance the practicality and feasibility of electrochemical biosensors for bacteria detection in wastewater treatment, which inspired the use of nickel (Ni) in the present study.

The current work aims to develop a standalone, portable electrochemical microbial sensor (EMS) for the electrochemical detection of *E. coli* in wastewater treatment using Ni oxidation. This amperometric EMS utilizes constant potential oxidation of nickel hydroxide (Ni(OH)_2_) to nickel oxyhydroxide (NiOOH) on a rotating disk electrode (RDE) in alkaline media to quantify *E. coli* in synthetic solutions ranging from 10^4^ to 10^10^ colony-forming units per milliliter (CFU/ml). Nickel electro-oxidation can be performed locally at the electrode surface on demand, aiding the sensor's longevity, and eliminating enzyme inactivation. The RDE technique is applied to introduce controlled, consistent mass transport of hydroxyl ions and *E. coli* to the Ni electrode surface, providing some insight into the detection mechanism.

## 2 Materials and methods

### 2.1 Experimental setup

**Fig. 1:**
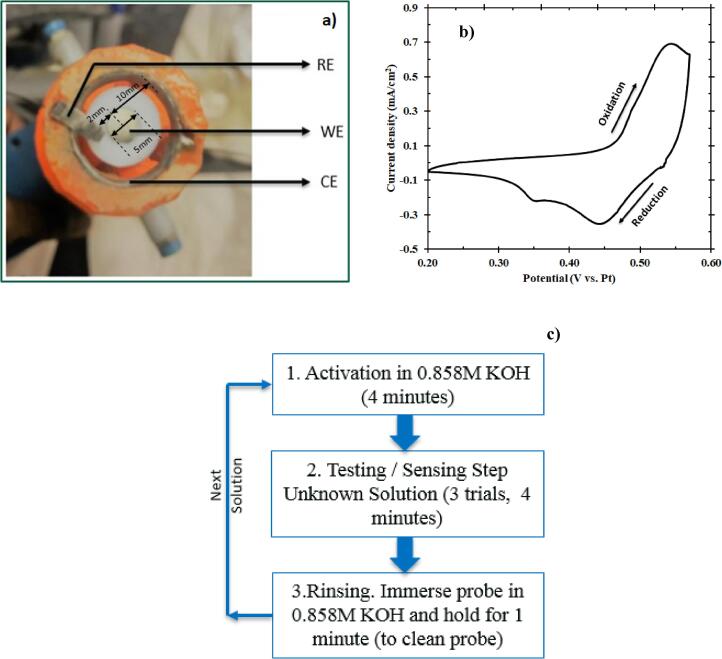
(a) Three-electrode configuration of the probe (bottom view) where the working electrode is a Ni disk in the center surrounded concentrically by a Pt ring serving as the counter electrode. At the same time, a Pt foil was used as the pseudo-reference electrode and placed close to the working electrode. (b) Cyclic voltammogram of Ni (5th cycle) in 0.858 M KOH performed in a potential window of 0.20–0.57 V vs. Pt at a scan rate of 15 mV/s. 5th cycle was the sustained periodic cycle. (c) Standard operating procedure for the electrochemical microbial sensor. This procedure involves an activation step (CV) followed by the testing step (Chronoamperometry). It renders a current response from the sample; this is followed by a rinsing step to disinfect the probe before subsequent tests.

Potassium hydroxide pellets (KOH) were purchased from Fisher Scientific (lot# 164253, 85.8% assay). Ultrapure water (>18 MΩ) was used throughout testing. All electrochemical measurements were performed in a 100 ml beaker using a Gamry Reference 600 Potentiostat. A Ni foil (Pine Research Instrumentation Inc., AFED050P040NI) was the working electrode (WE), and two platinum (Pt) foils (ESPI Metals, 99.95%, CAS 7440-06-4) acted as the counter electrode (CE) and pseudo-reference electrode (RE) as shown in Fig. 1a. The Pt counter electrode (0.75″ × 0.4″ × 0.005″) was made into a ring and placed concentric to the working electrode. The Pt reference electrode (0.6″ × 0.1″ × 0.005″) was immersed in test solution close to working electrode. A 3D printed casing (ABS 2.85 mm orange, Lulzbot Taz 6) held the counter and reference electrodes. Ni wires (Alfa Aesar, 99.5%, 1 mm diameter) were the current collectors. A Miller® resistance spot welder (SSW-2020ATT) spot welded the Ni wires on the Pt foils at 9 A per second.

The Ni foil was encased in a Teflon sleeve and mounted to a Pine Instrument Company MSRX Speed Control Analytical Rotator. The Ni foil was polished using sandpaper (400 grit) to remove any impurities followed by polishing using alumina slurry (Electron Microscopy Sciences, 0.05 μm, Lot # 160517) on micro cloth until the electrode surface appears to be mirror-like*.* The preparation of test solutions involved diluting 1 ml of 0.858 M KOH in 99 ml of *E. coli* solution for testing.

### 2.2 Bacterial growth conditions

*E. coli* strain MG1655 was grown at 37 °C with shaking (200 rpm) in lysogeny broth (LB). Overnight (~16 h) cultures of *E. coli* were centrifuged at 10,000 rpm for 5 min and then resuspended in sterile water. Cultures were then added to 1000 ml of water for probe testing. Dilution of the water and *E. coli* mix provided a range of concentrations for testing with the sensor. All dilutions were then quantified by BacTiter-Glo™ assay (Promega), standard plate count (on LB agar), or both these techniques.

### 2.3 Bacterial enumeration.

Although the standard plate count technique initially quantified *E. coli* concentrations, there was enormous time loss before obtaining a numeric value for the concentrations. Hence, with an intent to quantify the concentrations faster, commercially available chemiluminescent BacTiter-Glo™ was sought to quantify the concentrations of bacteria in each solution. BacTiter™ solution (100 μl) and bacterial culture in water (100 μl) were mixed in a 96 well plate. Following this, 5 min of shaking occurred within a BioTek Synergy multi-plate reader for obtaining the luminescence reading. For optimization, three biological replicates of the *E. coli* strains were serial diluted and plated on agar plates to determine *E. coli* concentrations. The values from plate counting were compared to BacTiter-Glo™ luminescence values to generate a standard calibration curve. For all experiments presented in this paper, concentrations obtained from as-calibrated BacTiter-Glo™ served as the reference.

### 2.4 Cyclic voltammetry

The catalyst layer was formed by performing an activation step. Activation step comprised of cyclic voltammetry (CV) in a potential window of 0.20–0.57 V vs. Pt at a scan rate of 15 mV/s in an alkaline environment containing 0.858 M KOH [23]. Pt pseudo-reference electrode, as opposed to a traditional reference electrode, acted as the reference electrode with an intent to use the EMS for testing contaminated water, mimicking a field scenario, where the usage of traditional reference electrodes might fail due to risk of contamination. The sustained periodic cycle, shown in Fig. 1b, was achieved after five cycles per previously reported results [24]. Although this is the general procedure for catalyst formation before testing the sample, it has to be noted that a one-time conditioning of the nickel electrode (as purchased) was performed the first time it was used. A cyclic voltammetry for 300 cycles at the same conditions mentioned above was performed for aging the nickel surface to induce the formation of an active NiOOH layer [25], [26]. The anodic peak at 0.55 V vs. Pt was attributed to the one-electron oxidation of Ni(OH)_2_ to form NiOOH as represented in the forward reaction of Eq. (1). During the reverse scan, two different cathodic peaks formed from two different states of Ni(OH)_2_ [24]. The first cathodic peak at 0.45 V vs. Pt indicates the one-electron reduction of β-NiOOH to form β-Ni(OH)_2_ and the second, smaller cathodic peak at 0.35 V vs. Pt represents the one-electron reduction of γ-NiOOH to form α-Ni(OH)_2_ [24], [27], [28].

(1) $${\left(Ni(OH\right)}_{2}+{\mathrm{OH}}^{-}⇌NiOOH+H_{2}O+e^{-}$$

### 2.5 Chronoamperometry

Considering the prospect of Ni(OH)_2_ to electrochemically oxidize in alkaline solution, the experimental procedures, shown in Fig. 1c, were carefully developed to maintain consistent redox properties of the Ni(OH)_2_/NiOOH layer during testing and therefore, consistent experimental results. The Ni(OH)_2_ layer was initially formed by CV, as described above. The electrode was then immersed in a synthetic solution containing *E. coli* (99 ml) and KOH (1 ml of 0.858 M KOH). The final concentration of the synthetic solution (8.58 mM) was chosen so that the *E. coli* cell death was prevented from occurring. The working electrode was rotated at 1600 revolutions per minute (rpm) (refer Fig. S1a, b in Electronic supplementary information). The open-circuit potential (OCP) was monitored until reaching a steady value (60 s), suggesting that the surface Ni(OH)_2_/NiOOH layer was in equilibrium with the solution. Chronoamperometry was then used to measure the constant potential for nickel oxidation and reduction current. The Ni(OH)_2_ was oxidized at 0.58 V vs. Pt for 5 s to provide sufficient overpotential for NiOOH formation while avoiding excessive water electrolysis. Finally, the NiOOH was reduced at 0.10 V vs. Pt for 15 s to reform the Ni(OH)_2_ layer before subsequent activation and testing. A point to be noted here is that that the presence of KOH and the electrochemical reaction conditions caused no significant variation in the viability of *E. coli*, indicating neither cell death nor significant cell growth throughout the sensing procedure (refer Table S1 in Electronic supplementary information).

## 3 Results and discussion

### 3.1 Electrochemical sensor performance

**Fig. 2:**
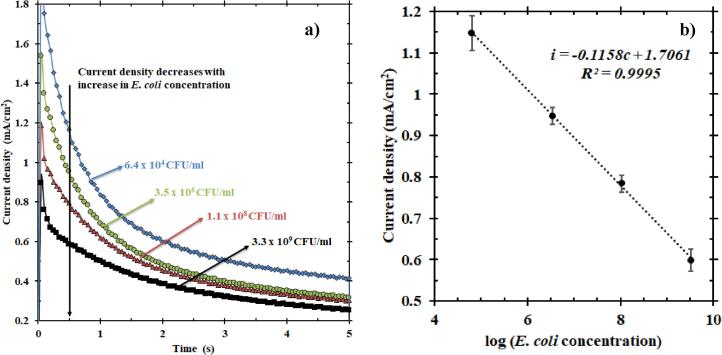
(a) Chronoamperometry plot representing the average of current densities of four samples with known *E. coli* concentrations over four trials; zoomed to show the 5 s of oxidation at a fixed potential of 0.58 V vs. Pt. The current density decreases with an increase in the concentration of *E. coli.* (b) The current density responses of these solutions at 0.5 s helped develop this calibration plot (fitted with least-squares linear regression). This plot shows the current density as a function of *E. coli* concentration in the logarithmic scale. The equation thus obtained is *i* (mA/cm^2^) = −0.1158*c* (mA/cm^2^) + 1.7061 (mA/cm^2^), where *i* is the current density and *c* is the log of *E. coli* concentration.

The sensor was tested using four known concentrations of *E. coli*: 6.40 × 10^4^ CFU/ml, 3.50 × 10^6^ CFU/ml, 1.10 × 10^8^ CFU/ml and 3.30 × 10^9^ CFU/ml. As shown in Fig. 2a, the presence of increasing *E. coli* concentrations in solution caused a significant decrease in the current density measured, consistent with previously reported data [29]. Each *E. coli* sample was tested four times to provide a reasonable standard deviation in the measured current density. A distinct separation in current density during constant potential oxidation was observed in less than 0.5 s. However, for convenience, the current density at 0.5 s was chosen to develop a calibration equation. Fig. 2b shows the concentration of *E. coli* (in logarithmic scale) as a function of its corresponding current density at 0.5 s. This testing using the rotating disk electrode revealed a highly linear relationship between current density and logarithm of *E. coli* concentration. Least squares linear regression fit the data, resulting in a calibration equation for the EMS at a high *R*^2^ value (*R*^2^ ~ 1*)*. The limit of detection was in the order of 10^4^ CFU/ml. In addition to testing these synthetic solutions (only *E. coli*), samples with *E. coli* in control solution (containing chemical components present in wastewater that could potentially interfere with EMS measurement) were also tested and the results are discussed in Section 1.3 of the Electronic supplementary information. Moreover, these chronoamperometric tests reported in Fig. 2a for a rotating disk electrode can be modeled by integrating transport equations, electroneutrality, material balance, and the charge transfer (electrokinetics of the NiOOH) reactions in the presence and absence of *E. coli*. The results can be combined with modeling discrimination techniques to provide a better understanding of the mechanism of the sensor and also for machine learning [30].

For validation, the same testing procedure was followed for the samples of unknown concentration (samples shown in Table 1 whose concentrations were blinded during testing and predicted by EMS using the calibration equation in Fig. 2b). The current density at 0.5 s was substituted in the calibration equation to find the logarithm of *E. coli* concentration. This value can be compared with the logarithm of *E. coli* concentration obtained from BacTiter-Glo™ to calculate the error in measurement. Table 1 shows the results obtained from testing solutions with unknown *E. coli* concentration.

**Table 1:** Quantification of unknown *E. coli* concentration in sample solutions.

Sample	Logarithm of *E. coli* concentration from BacTiter™	Logarithm of *E. coli* concentration from EMS	Relative Standard Deviation (%)	Percentage error vs. BacTiter™ (%)
1	9.57	9.71 ± 0.54	5.5	−1.5
2	8.04	8.98 ± 0.51	5.7	−11.7
3	7.26	7.94 ± 0.24	3.0	−9.4
4	6.57	8.12 ± 0.62	7.6	−23.6
5	5.47	6.32 ± 1.26	19.8	−15.7
6	4.38	6.03 ± 0.31	5.2	−37.8

While the detection limit of EMS does not reflect a drastic improvement over comparable electrochemical techniques, EMS can quantify *E. coli* concentrations close to the sophisticated BacTiter-Glo™ technique. It is worth noting that this detection and quantification is achieved by using relatively abundant electrode material (Ni), low response time for sensing the presence/absence of *E. coli*, low data acquisition/assay time, and formation of the fresh catalyst before every test. These advantages of EMS suggest that integration of this platform may have promise as a more economical and pragmatic alternative.

### 3.2 Specificity

**Fig. 3:**
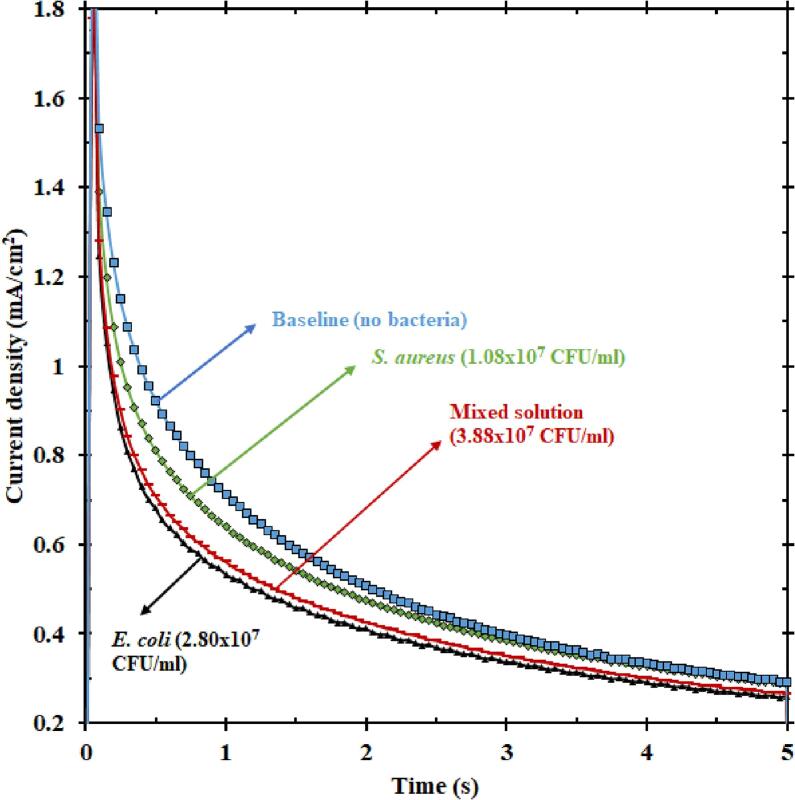
Plot showing the current responses (5 s of oxidation) of baseline KOH, only *E. coli*, only *S. aureus*, and a solution mixed with both *E. coli* and *S. aureus*. The current from the mixed solution is closer to that of *E. coli* than *S. aureus*, indicating that *E. coli* dominates the current response of mixed solution.

For examining the specificity of EMS in detecting *E. coli*, a solution of *E. coli* and a solution of the bacterium *Staphylococcus aureus* (*S. aureus*) were tested individually, and a mixed solution of these two bacteria was also tested. Although *S. aureus* is not a common bacterium found in the water or wastewater sources, the reason for choosing *S. aureus* was to check if gram-positive bacteria also had a response as investigated in other literature [29]. Interestingly, there was an electric current response from *S. aureus*. Nevertheless, the electric current from *E. coli* seemed to be much higher, as seen in Fig. 3. Also, the current response of the mixed solution almost traced *E. coli* current even when the total concentration of the mixed solution was higher than that of just *E. coli*. Even though the concentration of the mixed solution was 3.88 × 10^7^ CFU/ml, the concentration of *E. coli* was only 2.80 × 10^7^ CFU/ml. The current from mixed solution emphasizes that the current from *E. coli* was dominant compared to that of *S. aureus*, rendering *E. coli* specific current from the mixed solution. Such a dominant trend in terms of current has previously been reported in literature [29]. A potential reason for this dominance is due to the ability of *E. coli* to move towards the anode (nickel electrode) faster under applied electric field [31], thereby contributing to the electric current response faster than *S. aureus*. Another possible explanation could be the dynamic nature of *E. coli* in causing diseases in general*.* This gram-negative bacterium regulates more genes than *S. aureus* in a given time, thereby causing inflammation quicker than *S. aureus* [32]. In summary, the dynamic movement of *E. coli* towards the electrode leads to a current response overpowering the response from *S. aureus*, thus making this sensor turn electric current responses specific to that of *E. coli*. Interestingly, information of the chronoamperometry response can be evaluated a longer time, for example after 3 s, in which the response of *S. aureus* blends with the baseline. Our technique can leverage patterns at outer spatial times to discriminate among different type of microbes.

### 3.3 Hypothesized mechanism

**Fig. 4:**
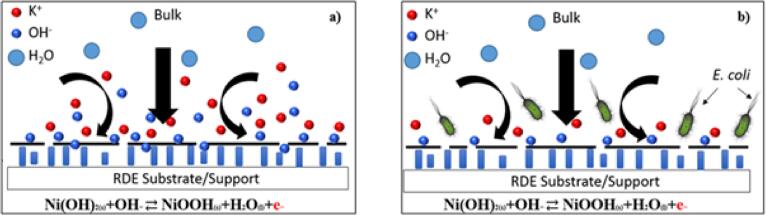
Schematic representation of the electrode/electrolyte interface in (a) absence of *E. coli* and (b) presence of *E. coli*. The presence of *E. coli* inhibits the diffusion of hydroxyl ions to the electrode surface leading to a depletion in the hydroxyl ions available for the Ni electro-oxidation to occur, thereby decreasing the current signal from the reaction.

The results obtained above provide some insight into the potential mechanism associated with the detection process. The hypothesis is that *E. coli* partly inhibited the diffusion of hydroxyl ions from forming NiOOH at the electrode surface (as shown in Fig. 4). This inhibition implies that there was a reduction in the availability of hydroxyl ions for the forward reaction in Eq. (1) to occur, resulting in inhibition of the rate of oxidation reaction and hence, reduced electron generation. Consequently, a reduction in current was observed during the constant potential oxidation of Ni(OH)_2,_ as discussed above. However, when the constant potential reduction proceeds first, the separation in the current density observed was negligible compared to oxidation, which indicated that *E. coli* more significantly influenced Ni(OH)_2_ oxidation. This influence leads us to believe that the distinction in oxidation current lies in the transport of hydroxyl ions to the surface. In the case of reduction, H_2_O, which is present at high concentrations near the surface, can readily be consumed to reduce NiOOH, suggesting that *E*. *coli* does not adsorb to the surface and potentially block active sites. On the other hand, the oxidation reaction proceeds with a limited concentration (8.58 mM) of OH^−^; therefore, the change in current must be from the hydroxyl ions’ ability to reach the surface and react (check also Section 1.4 in Electronic supplementary information). We hypothesize that perhaps the *E*. *coli* competes with OH^–^ in solution, either lowering the diffusivity or temporarily lowering the surface reaction sites during the oxidation reaction.

Further elucidation of the detection mechanism will allow for the development of more efficient reaction conditions and more effective electrode structures, which may lead to improved sensitivity and lower detection limits. Future work should be in the direction of applying this electrochemical detection platform to other forms of bacteria, which might give an insight worthy of using the probe on field at wastewater sources.

## 4 Conclusions

With shortage of drinking water being an environmental issue across the globe, it is expected that the remaining drinking water sources would not be available for half the world’s population by 2025 [1]. This would press people to start recycling wastewater to be used as drinking water. Hence, devices for water quality monitoring will become an essential household equipment like the current water filters. To meet the current industrial requirement and future household requirement for rapid and reliable microbial sensors, devices like the developed electrochemical microbial sensor that can detect and quantify the concentration of bacterium *Escherichia coli* would be the approach sought. The EMS overcomes the limitations of using enzymes or other biorecognition elements since fresh catalyst is formed on the electrode surface before every test. As highlights, the EMS can sense the presence/absence of *E. coli* within half a second and has a total assay time of 10 min. The detection limit of this sensor is of the order of 10^4^ CFU/ml. But, it is expected that modifications to the electrode surface and catalyst formation can lower the detection limit. Another noteworthy finding was that the current response from *E. coli* was dominant compared to *S. aureus.* This dominance, centered on the hypothesis that *E. coli* reached the electrode surface faster and negated the current from *S. aureus* in a solution mixed with both these bacteria, makes the sensor specific to *E. coli*.

*E. coli* was used as a model organism to begin the study. Future work will focus on enhancing the detection limit of EMS and assessing its use for other bacterial species in water and food. Detailed studies on spectroelectrochemical characterization could shed more light on the detection mechanism. Modeling the electrokinetics in combination with discrimination techniques and machine learning are also future areas of research for the extension of the sensor to other microorganisms. Apart from these, EMS can be integrated with machine learning and artificial intelligence to find different patterns at different time slots from the enormous data points collected. These studies could help completely automating the sensor and potentially finding patterns to differentiate the various organisms present in water and wastewater sources. Once integrated, these sensors can sense samples, starting from homes to remote locations, and the results could be stored on the cloud and obtained via a mobile phone application.

## Declaration of Competing Interest

The authors declare that they have no known competing financial interests or personal relationships that could have appeared to influence the work reported in this paper.
