# Supplementary material for: Rapid electrochemical detection of Escherichia coli using nickel oxidation reaction on a rotating disk electrode
Source: Chem Eng J. 2021 May 1;411:128453. doi: 10.1016/j.cej.2021.128453 (PMC7957341; doi:10.1016/j.cej.2021.128453)
Supplement: Supplementary data 1 [file mmc1.docx]

**Rapid electrochemical detection of *Escherichia coli* using nickel oxidation reaction on a rotating disk electrode**

Ashwin Ramanujam,^a,b^ Bertrand Neyhouse,^b^ Rebecca A. Keogh,^c^ Madhivanan Muthuvel,^b^ Ronan K. Carroll,^c^ Gerardine G. Botte^a^*

^a^ Chemical and Electrochemical Technology and Innovation Laboratory

Department of Chemical Engineering, Texas Tech University, Lubbock, TX 79401, USA

^b^ Center for Electrochemical Engineering Research

Department of Chemical and Biomolecular Engineering, Ohio University, Athens, OH 45701, USA

^c^ Department of Biological Sciences, Ohio University, Athens, OH 45701, USA

*Corresponding author email: gerri.botte@ttu.edu

**ABSTRACT**

This file contains additional information on the electrochemical microbial sensor (EMS). Information regarding the operation and stability of EMS are included to show how experiment conditions were determined to reduce the mass transfer limitations and to get reproducible results. For investigating the applicability of this sensor in wastewater, a control solution containing major chemical components found in wastewater was prepared and tested for *E. coli* presence. Moreover, additional evidences for the hypothesis proposed in the paper indicated that the *E. coli* is competing with the hydroxyl ions at the interface, hindering them from reacting with nickel substrate; thereby resulting in a drop in current.

- 1. *Operating Conditions and Stability of EMS*

We initially observed that the sensing procedure depended significantly on the interaction of *E. coli* with the surface of the nickel electrode, indicating that transport of *E. coli* to the interface was an important step to be integrated with the procedure. Hence, the rotating disk electrode was introduced to minimize the mass transport limitations and facilitate the controlled flow of *E. coli* and diffusion of hydroxyl ions from the bulk to the interface. In order to determine optimal conditions, experiments were conducted setting the rotation speed of the RDE at 100, 400, 900, 1600 and 2500 rpm. The results of Chronoamperometry tests (Figure S1 a, b for 100 and 1600 rpm, respectively) at these speeds reveal that with increase in rotation speed, the mass transport limitations were decreased which resulted in better separation in current densities of different *E. coli* concentrations. The separation in current density between a baseline solution (only KOH and no *E. coli*) and solution containing 1.7 x 10^7^ CFU/ml *E. coli* at 100 rpm (Figure S1 a) was very low. However, at a higher rotation speed of 1600 rpm (Figure S1 b), there was increased separation in current density between the same samples. At 2500 rpm, the data show high levels of noise which could have been caused by a variation in the flow pattern from laminar flow. Based on these results, the rotation speed for EMS was fixed at 1600 rpm.

The stability and reproducibility of the EMS were tested on four random days (Day 1, 4, 16, 19) over a period of three weeks, in baseline KOH solution (99 ml DI water + 1 ml 0.858M KOH) without any *E. coli*. The current density response at 0.5 seconds in baseline KOH solution was measured every time before a solution with *E. coli* was tested, to make sure that the locally formed catalyst produced similar response. It can be observed from Figure S1 c that the current density response of EMS in baseline KOH was consistent without any major fluctuation. Even the minor changes in the current density values could have well been due to the error propagation from the concentration of stock KOH prepared manually on that day. The values plotted are an average of four to six baseline KOH samples tested on the corresponding days. The standard deviations of these samples on each day are minimal, indicating that the experiments were reproducible with minimal error. Moreover, the relative standard deviation of all the samples from these four days is 13%, indicating that there is no major variation in the reproducibility of results in baseline KOH. To support this case, the current density values of *E. coli* solutions on those four days (3.9x10^6^, 5.6x10^6^, 3.0x10^6^ and 1.8x10^7^ CFU/ml respectively) were compared to their respective baseline KOH values and from Figure S1 c, it can be seen that the current density data points of *E. coli* solutions (triangles) do not overlap with the deviation in current density data point from baseline KOH. This indirectly revealed that the surface of electrode was stable before the samples were tested. Since the current density response in KOH was similar, further tests were performed without any checks being performed in baseline KOH. To avoid the variation in baseline KOH current, it is recommended to use commercially available, standardized KOH solutions.


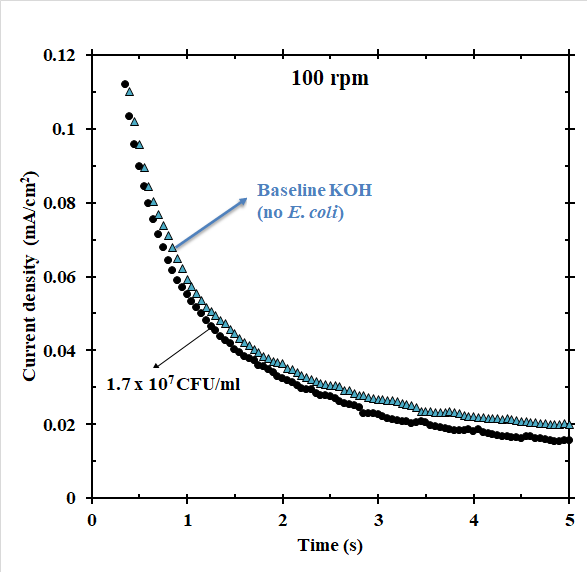

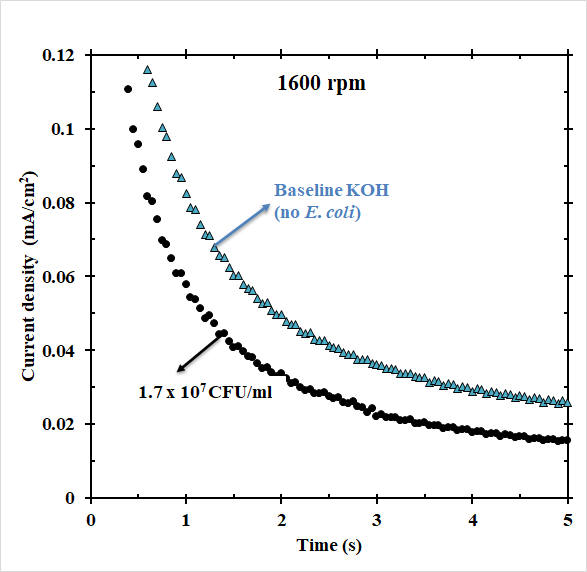


**b)**

**a)**


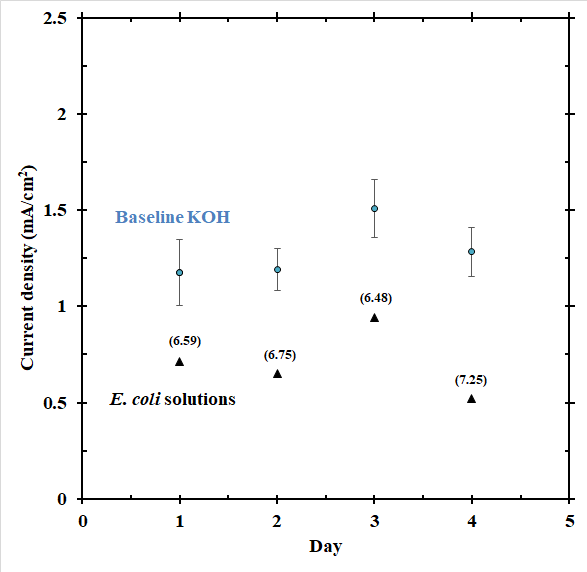


**c)**

**Figure S1.** Results of Chronoamperometry tests performed at rotation speeds of **a)** 100 rpm and **b)** 1600 rpm. The separation in current density at 1600 rpm is clearly higher than that observed at 100 rpm owing to improvements in mass transfer kinetics at the higher rotation speed. **c)** Plot showing the stability of baseline KOH (circles) over four different days. The data points in triangle represent the current density values obtained from *E. coli* solutions of concentrations ranging between 6.48 to 7.25 (logarithmic values in bracket), depending on the day they were tested. Clear separation in electric current between baseline and *E. coli* solutions is observed irrespective of any fluctuations in baseline KOH concentration.

- 1. *E. coli viability during measurement and rinsing*

In addition to this stability measurement, experiments for testing the viability of *E. coli* before and after the sample testing were done. The intention of these tests was to ensure that the viability of *E. coli* was not lost due increase in pH of test solution (pH~12) with addition of KOH. Table S1 shows the concentrations obtained from BacTiter-Glo^TM^ assay before and after testing was done with EMS. There is no reduction in the concentration of *E. coli*. The minor increase in concentration of *E. coli* can be attributed to the multiplication of this bacteria within the span of completing the experiment or the variability in standard plate count experiments. However, it should be noted that there was no *E. coli* present in the sample when the pH of solution was increased to ~14. Hence, by controlling the pH of rinse solution, the probe was disinfected between subsequent tests to eliminate carryover of *E. coli*.

**Table S1. Concentration of samples before and after testing showing evidence for *E. coli* viability during the testing procedure**

| **Sample No.** | **Sample taken before / after testing** | **Luminescence from BacTiter-Glo^TM^** **(RLU)** | ***E. coli* concentration**  **(CFU/ml)** |
| --- | --- | --- | --- |
| 1 | Before | 14267 | 1.93 x 10^8^ |
|  | After | 14414 | 1.98 x 10^8^ |
| 2 | Before | 433 | 2.40 x 10^5^ |
|  | After | 480 | 2.90 x 10^5^ |

- 1. *Test with Control Solution*

Experiments with synthetically prepared wastewater solution (control solution) containing *E. coli* in presence of phosphates, nitrates, ammonium sulfate, and urea were performed. The intention behind these experiments was to observe any possible interference from the mentioned chemical compounds that would potentially be present in wastewater stream. For this purpose, *E. coli* solution of concentration 8.4 x 10^9^ CFU/ml was mixed in a solution containing 400ppm Ammonium sulfate, 1,200ppm Sodium nitrate, 100ppm Sodium di-hydrogen phosphate, 12,000ppm Glucose, and 4,000ppm Urea. The pH of the control solution was 11.95 after addition of KOH. This solution was tested with EMS and the corresponding result can be seen in Figure S2 a. It can be noticed that there was a clear separation in comparison to the control solution without *E. coli* (baseline). This result suggests that the addition of KOH to the control solution (increase in pH) nullifies the effect of all other components present in that solution. It was only the *E. coli* that caused hindrance to the nickel electrochemical oxidation reaction resulting in current drop.

In another experiment, we found the electric current from an *E. coli* sample to be overlapping with the baseline current response of control solution in absence of *E. coli* as seen in Figure S2 b. This result had us validate the sample also with standard plate count technique where there was no *E. coli* growth on the plates. The inactivation could have been a result of unfavorable temperature or storage of the sample prior to testing. The electric current from this dead *E. coli* solution traced the current of baseline control solution without *E. coli*, meaning EMS was specific in detecting live *E. coli* in the samples.


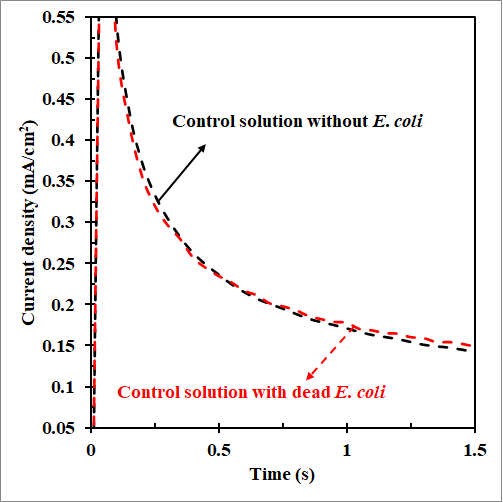

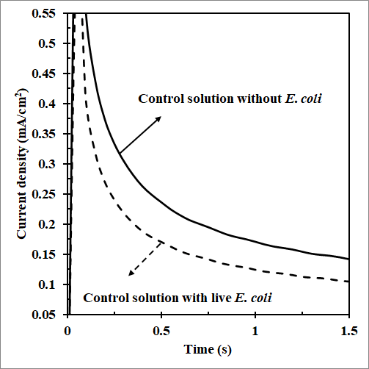


**Figure S2. a)** Chronoamperometry showing a clear current separation between control solution without *E. coli* and control solution with *E. coli* implying that *E. coli* can lower the current from Ni electrooxidation even in the presence of chemical constituents. **b)** Plot showing evidence that there was no current separation at all between the control solution without *E. coli* and control solution with dead *E. coli*. These two plots prove that the sensor only responds to signals from live *E. coli*.

- 1. *Evidence for Hypothesis*

A possible evidence to this hypothesis was also observed more recently in the cyclic voltammetry (CV) data obtained during the activation step mentioned in figure 1c of paper. Gold (Au) reference electrode was used for these experiments instead of platinum. It is well known that Pt can used as reference electrode in scenarios where conventional reference electrodes cannot be used.[1] In EMS, conventional reference electrodes would have failed if there were to be exposed to wastewater stream. Hence, Pt was chosen for its relatively inert nature. But, over time it was observed that there was shifts in the cyclic voltammograms of Ni electrooxidation. So, for these experiments, Pt was replaced with another stable electrode, Au.[2] As mentioned in section 2.4 of the article, the Ni electrode was activated in KOH solution before the testing step. From Figure S3 a and b, it can be noticed that the CV after testing the probe in a bacterial solution has shifted positively compared to the CV before testing bacterial solution (after testing baseline KOH). This indicates that the rate of formation of NiOOH was slower when testing the solution with bacteria compared to baseline KOH solution. Hence, the NiOOH formation was at a higher potential in the subsequent CV. This indirectly means that there was a reduction in OH^-^ ions available for forward reaction of equation 1 due to the presence of bacteria, resulting in lower NiOOH formation. This evidence for the mentioned hypothesis can further be studied in detail in the future by using microkinetics approach used for modeling the RDE based systems.[3] Studying the kinetics by modeling this system could help validate this hypothesis and illuminate the phenomena occurring at the interface.


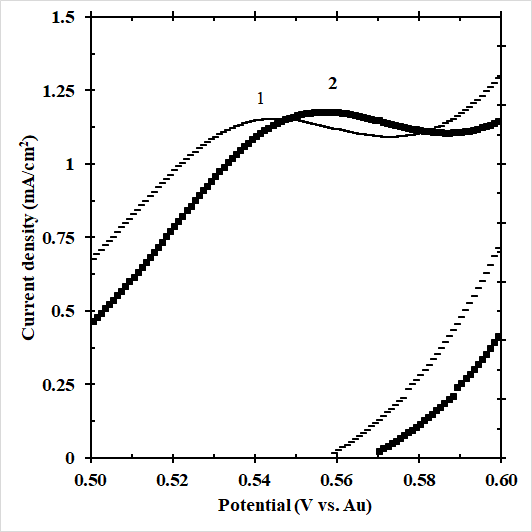

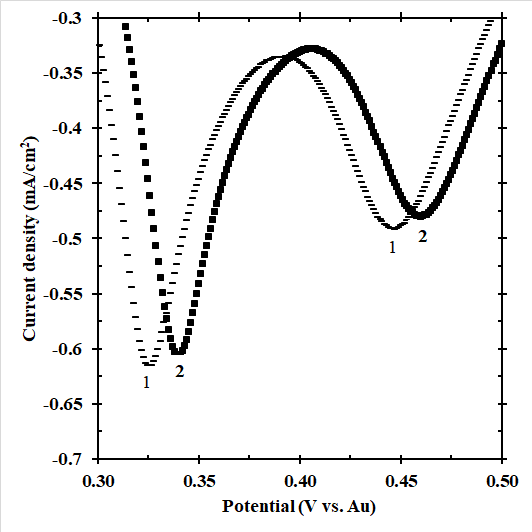


**b)**

**a)**

**Figure S3.** Positive shift of cyclic voltammetry after testing 1.62 x 10^8^ CFU/ml (2) as compared to the cyclic voltammetry before testing it (1) indicating that there was hindrance to NiOOH formation during the testing of bacterial solution. **a)** shows the oxidation peak of CV while **b)** shows the reduction peaks of CV

**References:**

[1] B.K.K. Kasem, S. Jones, Platinum as a Reference Electrode in Electrochemical Measurements, Platin Met Rev. 52 (2008) 100–106. https://doi.org/10.1595/147106708X297855.

[2] S. Solchenbach, D. Pritzl, E.J.Y. Kong, J. Landesfeind, H.A. Gasteiger, A Gold Micro-Reference Electrode for Impedance and Potential Measurements in Lithium Ion Batteries, J. Electrochem. Soc. 163 (2016) A2265–A2272. https://doi.org/10.1149/2.0581610jes.

[3] L.A. Diaz, G.G. Botte, Mathematical modeling of ammonia electrooxidation kinetics in a Polycrystalline Pt rotating disk electrode, Electrochimica Acta. 179 (2015) 519–528. https://doi.org/10.1016/j.electacta.2014.12.162.
